# The monoclonal antibody for discrimination of natural honey against artificial honey

**DOI:** 10.1002/fsn3.821

**Published:** 2018-10-25

**Authors:** Taeri Joe, Eunbee Jeong, Gaeun Yoo, Sejin Ahn, Hae‐ik Rhee, Deug‐Chan Lee

**Affiliations:** ^1^ Department of Biomedical Technology Kangwon National University Chuncheon Korea; ^2^ Institute of Bioscience and Biotechnology Kangwon National University Chuncheon Korea

**Keywords:** artificial honey, ELISA, monoclonal antibody, natural honey

## Abstract

**Background:**

Honey is a natural product used as food, medicine, or cosmetics for very long time and is made by bees. Honey contains various components such as sugar, protein, minerals, and vitamins. Honey is made by *Apis cerana* or *Apis mellifera*, which commonly has major royal jelly proteins (MRJPs) as a major protein. To discriminate between natural honey (NH) and artificial honey (AH), many researchers tried method of physicochemical analysis. However, the analysis results were ambiguous and not stable.

**Results:**

We have produced a monoclonal antibody that recognizes MRJPs of honeys in common. Monoclonal antibody has advantage such as accuracy, sensitivity, and stability as the standard. The specificity and affinity of produced antibody were measured by western blotting and enzyme‐linked immunosorbent assay. As a result, this monoclonal antibody specifically recognized MRJPs of NH and did not recognize AH which has not including MRJPs.

**Conclusion:**

Natural honey could be able to distinguish from AH accurately by using this monoclonal antibody. Also, this method could be commercially applicable.

## 1 INTRODUCTION

Honey is used for broad applications including food additives and cosmetics in worldwide (Ediriweera & Premarathna, 2012). Honey constituents and their biological activities have been investigated. Main constituents of natural honey (NH) are sugars, minerals, proteins, and other organic acids (Alvarez‐Suarez, Tulipani, Romandini, Bertoli, & Battino, 2010). NH has been used as traditional medicine for a long time (Eteraf‐Oskouei & Najafi, 2013).

In the ecology of honeybee, queen larva is fed solely with royal jelly, a secretion from the hypopharyngeal gland of worker bees (Mykola, 1970). The most abundant proteins in royal jelly, comprise about 10% of royal jelly, are termed as major royal jelly proteins (MRJPs). MRJPs have been found not only in royal jelly also in other natural products of honeybee. Different types of MRJPs were identified in different honey types and pollen stores of honeybee colony (Schmitzová et al., 1998).

Honey is generally classified by two ways. One is kind of honeybee species, *Apis cerana* or *Apis mellifera*. The other is nectar source, for instance acacia honey, chestnut honey, and multifloral honey. In the markets, the honey that is extended using sugar or is mixed with artificially compounded honey also has been sold. Therefore, it has been required that the way to determine the NH from artificial honey (AH). Although it has been reported to the NH‐specific molecular constituents such as 5‐hydroxymethyl‐2‐furaldehyde (HMF), discrimination between natural and artificially synthesized honey still remains as a challenge. Several approaches were developed for the detection of honey adulteration, which is the addition of cane or beet sugars or sugars obtained from starch hydrolysis (Chen et al., 2011; Paradkar & Irudayaraj, 2002).

However, such approaches are not suitable for industrial or commercial applications because of the method using analytical chromatography. Instead, for the discrimination between natural and artificially synthesized honey, enzyme‐linked immunosorbent assay (ELISA) method based on the monoclonal antibodies (mAbs) is more suitable in practical applications. For these reasons, we developed specific and sensitive mAbs against major honey protein based on the similarity of MRJPs sequence from *A. cerana* and *A. mellifera* (Won, Lee, Ko, Kim, & Rhee, 2008). As has been mentioned, MRJP is a protein of honeybee origin. As honeybees ingest NH, they return to the honeycomb and spit MRJPs out. These proteins are stored with honey and contained in a certain amount in honey. Therefore, NH contains theses MRJPs, but it is not contained in the AH made from yeast invertase. Therefore, it is possible to distinguish between NH and AH depending on the presence or absence of MRJPs (Buttstedt, Moritz, & Erler, 2014; Won et al., 2008). In addition, it is anticipated that when the research for establishing the standard of these protein amounts in NH is proceeded more precisely, it will be possible to discriminate mixed honey made by mixing AH with NH.

## 2 MATERIALS AND METHODS

### 2.1 Sample preparation

Natural honeys made by *A. cerana* and *A. mellifera* were purchased at the local market in South Korea. They were dialyzed using dialysis membrane, centrifuged to remove the pollen. The supernatant was concentrated by freeze drying and used as an antigen for antibody preparation. A honey which has been used as antigen in western blotting and ELISA is centrifuged to remove the pollen, and the information is summarized in Table 1. AH samples were prepared as described by the study of Tonks et al. (2003).

**Table 1:** The list of experimentally used natural honeys

No.	Harvesting area	Species	No.	Harvesting area	Species
NH1	Yeongwol‐gun, Gangwon‐do	*Apis cerana*	NH11	Jeongseon‐gun, Gangwon‐do	*Apis mellifera*
NH2	Jeongseon‐gun, Gangwon‐do	NH12	Jeongseon‐gun, Gangwon‐do
NH3	Jeongseon‐gun, Gangwon‐do	NH13	Sangju‐si, Gyeongsangbuk‐do
NH4	Jeongseon‐gun, Gangwon‐do	NH14	Hoengseong‐gun, Gangwon‐do
NH5	Jecheon‐si, Chungcheongbuk‐do	NH15	Hoengseong‐gun, Gangwon‐do
NH6	Chungju‐si, Chungcheongbuk‐do	NH16	Daejeon Metropolitan City
NH7	Pocheon‐si, Gyeonggi‐do	NH17	Yangyang‐gun, Gangwon‐do
NH8	Jecheon‐si, Chungcheongbuk‐do	NH18	Yangyang‐gun, Gangwon‐do
NH9	Yangpyeong‐gun, Gyeonggi‐do	NH19	Yangyang‐gun, Gangwon‐do
NH10	Yangpyeong‐gun, Gyeonggi‐do	NH20	Yangyang‐gun, Gangwon‐do

### 2.2 Monoclonal antibody production

The female BALB/c mice were immunized with 100 μg of honey diluted in 100 μl PBS and complement Freund's adjuvant mixture (Cat# F5881; Sigma). As an immunogen, honey harvested at Macheon in Jiri‐Mountain and commercially available acacia honey were used as *Apis cerena* origin and *A. mellifera* origin, respectively. After 2 weeks, same honey and incomplement Freund's adjuvant (Cat# F5506; Sigma) mixture were immunized. Two more injections were existed. One week after the last injection, the mice were sacrificed. The mouse spleen cells were fused with mouse myeloma cells, SP2/0 at the ratio of 1:10 by polyethylene glycol (PEG1500; Roche). Then, the cells were cultured into 96‐well plates by hypoxanthine‐aminopterin‐thymidine medium (HAT medium, Gibco) and incubated with 5% CO_2_ at 37°C. About 1 week later, HAT medium was removed and changed to HT medium (Gibco).

### 2.3 Antibody purification

The antibody production clone was incubated for 3 days, and the supernatant was collected. The supernatant was spin downed to get rid of cell debris at 3,100 *g* for 5 min. Then, the ammonium sulfate was added to supernatant in the amount to 55% from supernatant weight. This mixture was stirred for overnight slowly. After that, mixture was spin downed at 4,000 rpm for 30 min. Pellet was resuspended in 30 ml cold PBS with 0.05% NaN_3_ and spin downed for at 3,000 rpm for 10 min to remove all particles. The supernatant was moved to dialysis membrane and dialyzed against 1 L of cold PBS, 3 times for 9 hr, and change PBS every 3 hr.

### 2.4 Enzyme‐linked immunosorbent assay

To determine the affinity of Ab, indirect ELISA was performed. The antigens were prepared that mixture of four samples of AH was blended with mixture consist of 10 samples of *A. cerana* origin honey or 10 samples of *A. mellifera* honey (Table 1) in the ratio one to one. All antigens were coated with carbonate coating buffer (pH 9.6) for overnight at 4°C, washed by PBS‐T, and blocked with 1% BSA (Hyclone) blocking buffer. After washing, the TY antibody was reacted for 1 hr. Washing again, and goat anti‐mouse IgG HRP (sc‐2005; Santa Cruze) as secondary antibody was treated for 1 hr. After three times washing with PBS‐T, ABTS (Sigma) with 0.03% H_2_O_2_ was added and incubated at 37°C for 20 min. When sufficient color was developed, absorbance was measured at 405 nm.

### 2.5 Western blotting

Western blotting was used to determine the recognized patterns of honey proteins by antibody which is recognizing both native bee honey and foreign bee honey. A mixture of NH with AH in various ratios was loaded to 10% SDS‐PAGE gel and transferred to a PVDF membrane (GE healthcare). The membrane was blocked with 1% skim milk (Sigma) and incubated with TY antibody. Washing three times, membrane was incubated HRP‐conjugated goat anti‐mouse IgG (sc‐2005; Santa Cruze). After washing, the membrane was reacted with ECL substrate and exposed to film.

## 3 RESULTS AND DISCUSSION

### 3.1 Comparing the constituents of honey samples

Table 2 shows physicochemical analysis of NH and AH. Physicochemical analysis categories were contents of water, sucrose, invert sugar, HMF, and artificial sweetener. In all categories, NH and AH were not distinguished by physicochemical analysis. In this result, HMF of AH was measured as significantly high. We thought that this difference is induced because AH was heated in the process of water removing. If the water was eliminated in the condition of decompression, it would not be heated.

**Table 2:** Results of physicochemical analysis from NH and artificial honey (AH)

Honey		*n*	Water (%)				Sucrose (%)			
			Ave.	*SD*	Min.	Max.	Ave.	*SD*	Min.	Max.
NH	*Apis cerana*	55	18.1	1.71	14.6	23.7	1.81	4.39	0.00	20.44
	*Apis mellifera*	23	19.0	1.99	16.5	>25.0	0.45	1.54	0.00	7.46
AH		4	24.1	0.97	22.7	24.8	1.0	0.40	0.63	1.38
Total		82	18.62	2.2	14.6	>25.0	1.39	3.72	0.00	20.44

Honey		*n*	Invert sugar (%)				HMF (mg/kg)			
			Ave.	*SD*	Min.	Max.	Ave.	*SD*	Min.	Max.
NH	*A. cerana*	55	67.67	9.37	44.53	86.11	6.20	14.70	0.00	98.82
	*A. mellifera*	23	64.89	3.50	59.41	75.28	35.23	84.20	1.05	316.37
AH		4	67.50	2.67	63.71	69.62	251.6	6.23	247.22	260.85
Total		82	66.89	7.98	33.61	86.11	26.32	69.82	0.00	316.37
Artificial sweetener was not detected in all samples.										

*Note*

HMF: 5‐hydroxymethyl‐2‐furaldehyde.

### 3.2 Development and characterization of monoclonal antibodies

To select the hybridoma producing the high sensitive and specific mAbs against major honey proteins, ELISA screening was conducted. Based on the results of ELISA, we selected TY1 and TY2 hybridoma clones that showed more specific recognition of major honey proteins made by *A. cerana* and *A. mellifera* than other antibodies in both colorimetric signals and optical density of 405 nm. Next, we tested the isotype of the antibodies produced by TY1 and TY2 clones. As a result, we confirmed that the antibodies produced by TY2 clone had an IgG1 kappa isotype (Table 3) but other antibodies produced by TY1 clone were mixture of IgG1 and IgM kappa isotypes (data not shown). Therefore, the antibodies produced by TY2 clone were used for following experiments such as Western blot analysis and ELISA.

**Table 3:** Characteristics of the developed monoclonal antibody

Name of monoclonal antibody (mAb)	The type of recognizable antigen by mAb	Isotype of mAb	Type of heavy chain	Type of light chain
TY	Honey made by *Apis cerana*	IgG1	γ1	κ
Honey made by *Apis mellifera*			

### 3.3 Specificity test of the monoclonal antibody in ELISA

**Figure 1:**
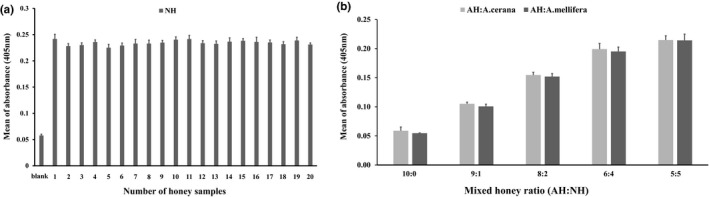
Specificity of monoclonal antibody using ELISA. (a) TY monoclonal antibody we produced had the similar level of absorbance at 405 nm in natural honeys (NHs) made by *Apis cerana* or *A. mellifera*. (b) In mixed sample with NH and artificial honey (AH) depending on the ratio, TY antibody specifically recognized as much as included NH rate. Results are presented as the mean ± *SD* of three independent experiments. ELISA: enzyme‐linked immunosorbent assay

To evaluate the specificity of mAbs, we conducted ELISA. Selected NHs were used as an antigen and same Ab, and TY was used for detection Ab. This result was showed that TY antibody equally detected both NH made by different species (Figure 1a). Then, whether this method is applicable to the detection of adulteration in mixed honeys or not, we mixed NH with AH in different proportions and used as antigen. TY antibody was used for detection antibody (Figure 1b). As shown in Figure 1b, the mAb against major honey proteins recognized in proportion with the amount of the major proteins of NH, whereas artificially synthesized honey was not recognized by the TY antibody, as expected. Therefore, these data suggested that the ELISA method based on anti‐major honey protein mAb, developed in this study, is applicable to determination whether the honey was naturally pure or artificially mixed. In terms of the y axis in Figure 1b, the absorbance value between unmixed NH and NH mixed with AH may seem like not much different, but it is the character of enzyme‐conjugated antibody reaction. That is because the calibration curve by using enzyme‐conjugated secondary antibody forms the sigmoidal curve depending on the concentration of the antigen, and this absorbance value is meaningful for discrimination the pure honey from the artificially mixed honey.

### 3.4 Antigen recognition of the monoclonal antibody on Western blot analysis

**Figure 2:**
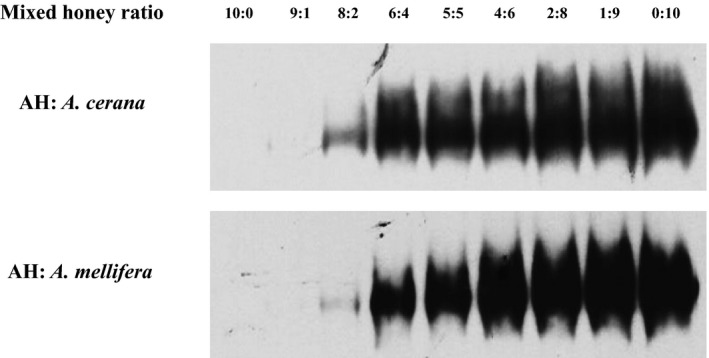
Sensitivity of monoclonal antibody to mixed honey samples in western blotting. Though natural honeys (NHs) were mixed with artificial honey (AH) in varying ratios, TY monoclonal antibody displayed specific recognition to NHs compared to AH. Western blotting signals became gradually stronger as proportion of NH. Also, there was no significant different in band size or darkness between NH made by different species

Subsequently, to validate the sensitivity of the antibodies, we performed SDS‐PAGE followed by Western blot analysis. Samples that are mixed NH with AH by the ratio were loaded (Figure 2). As shown in Figure 2, band signals were strengthened in accordance with amount of the loaded NH. Therefore, these data suggested that this TY antibody sensitively recognizes the major proteins of NH.

## 4 CONCLUSIONS

The present study developed the high sensitive and specific mAb against major honey proteins produced by *A*. *cerana* and *A. mellifera*. The specificity and sensitivity of the mAb were validated using ELISA and Western blot analysis. In the specificity test, although this mAb showed similar recognition to both NHs made by *A*. *cerana* and *A. mellifera*, distinguishment NHs from artificially synthesized honey was definite. Therefore, it is the possible usage of ELISA method practically for determination whether the honey was naturally pure or artificially mixed with other adulterations. Taken together, we expected that whether the honey derived from honeybees or the honey blended with adulterations can be accurately and rapidly determined by immunological method, ELISA based on the anti‐major honey proteins mAb (TY). Also, since the supply of antibody for discrimination is highly stable, it could be the appropriate standard in food industry.

In Korea, however, the amount of honey produced by *A. cerana* and *A*. *mellifera* differs depending on the amount of honey that needs to be distinguished (Rhee & Kim, 1996). Therefore, an antibody recognizing each of the honey produced by *A. cerana* or *A*. *mellifera* should be produced and be distinguishable to help the accurate honey consumption.
